# The Effect of Subcutaneous Saline Irrigation on Wound Complications After Cesarean Section: A Systematic Review and Meta-Analysis of Randomized Controlled Trials

**DOI:** 10.7759/cureus.62152

**Published:** 2024-06-11

**Authors:** Wardah Albzea, Lolwa Almonayea, Mooza M Alzayed, Abdullah M Alharran, Hanaa F Alrashidi, Sarah Alenezi, Hamdah Hadi

**Affiliations:** 1 Internal Medicine, Faculty of Medicine, Alexandria University, Alexandria, EGY; 2 Obstetrics and Gynecology, Kuwait Institute for Medical Specializations, Kuwait City, KWT; 3 College of Medicine, Arabian Gulf University, Manama, BHR; 4 Medicine and Surgery, Kuwait Institute for Medical Specializations, Kuwait City, KWT; 5 Medicine and Surgery, Farwaniya Hospital, Ministry of Health, Kuwait City, KWT

**Keywords:** surgical site infection (ssi), hematoma, systematic review and meta-analysis, normal saline, cesarean section (cs)

## Abstract

Subcutaneous (SC) saline irrigation was reported as a feasible and cost-effective procedure to prevent cesarean section (CS) surgical site complications. We aim to investigate the efficacy of SC saline irrigation to prevent CS surgical site complications. A systematic review and meta-analysis were conducted synthesizing evidence from randomized controlled trial (RCT) studies obtained from PubMed, Embase Cochrane, Scopus, and Web of Science from inception to March 2024. Pooled outcomes included wound complications (superficial surgical site infections (SSI), hematoma, seroma, and wound separation) and operative time. We used RevMan v.5.4. (The Cochrane Collaboration, Oxford, UK) to report dichotomous outcomes using risk ratio (RR) and continuous outcomes using mean differences (MD) with a 95% confidence interval (CI). Five RCTs with 4,025 patients were included. Four studies had a low overall risk of bias and only one trial with some concerns about selection bias. There was no difference between SC saline irrigation and no irrigation regarding the incidence of superficial SSI (five RCTs, RR: 0.72 with 95% CI [0.47, 1.10], P = 0.13), seroma (four RCTs, RR: 0.73 with 95% CI [0.32, 1.65], P = 0.45), wound separation (four RCTs, RR: 0.66 with 95% CI [0.36, 1.24], P = 0.2), and operative time (four RCTs, MD: -1.26 with 95% CI [-5.14, 2.62], P = 0.52). However, SC saline irrigation significantly decreased the incidence of hematoma (three RCTs, RR: 0.54 with 95% CI [0.45, 0.65], P = 0.00001). SC saline irrigation of the surgical site after CS was not effective in preventing the incidence of superficial SSI, seroma, or wound separation, while only preventing the incidence of hematoma.

## Introduction and background

Cesarean section (CS) is the most prevalent obstetric procedure, accounting for 60% of deliveries in some countries [1,2]. Also, CS prevalence has been swiftly increasing during the last decades, particularly in high-income countries [3,4]. CS can lead to various complications, including surgical site complications, which can be classified as infectious or non-infectious. The rate of CS surgical site complications was reported to vary from 3% to 30% [5-7]. Infectious complications, such as superficial surgical site infections (SSI), or non-infectious complications, such as hematoma, seroma, or wound separation, present a challenging issue during puerperium [7]. CS surgical site complications can significantly increase post-CS maternal morbidity and burden on healthcare services [8].

Multiple strategies have been investigated to prevent the incidence of CS surgical site complications [9], including prophylactic antibiotics, skin preparation, and subcutaneous (SC) irrigation [8,10]. SC irrigation was investigated using multiple agents. Irrigation of SC tissue using either topical antibiotic, povidone-iodine, or saline has been reported to prevent superficial SSI post-CS [8,11]. Normal saline (NS) is the safest solution used in SC irrigation; therefore, it is the most prevalent in obstetric practice [8]. Also, it was reported to be a feasible and cost-effective procedure to prevent CS surgical site complications [8,12].

However, a recent meta-analysis investigating the efficacy of SC saline irrigation after abdominal surgeries reported that SC saline irrigation was not effective in preventing SSI or decreasing the length of hospitalization [9]. Furthermore, multiple randomized controlled trials (RCTs) have been recently published, with conflicting results regarding SC saline irrigation post-CS [7,13,14]. Therefore, we aim in this systematic review and meta-analysis to investigate the efficacy of SC saline irrigation post-CS to prevent post-CS surgical site complications.

## Review

Methodology

The conduct of this systematic review and meta-analysis adhered rigorously to the guidelines outlined in the Preferred Reporting Items for Systematic Reviews and Meta-Analysis (PRISMA) [15] and the Cochrane Handbook of Systematic Reviews and Meta-Analysis [16].

Sources of Data and Search Methodology

We systematically searched electronic databases, including PubMed, Web of Science, Scopus, Cochrane CENTRAL, and Google Scholar until March 18, 2024. The following search strategy was used [(“cesarean section” OR “CS” OR “C-section” OR “cesarean delivery” OR “abdominal delivery”) AND (saline* OR NS OR “normal saline”OR “sodium chloride”) AND (irrigat* OR lavage OR “wound irrigat*” OR “wound infiltrat*” OR “subcutaneous irrigat*” OR “subcutaneous infiltrat*”)] without any search limits or filters. The detailed search results for each database are detailed in Table 1.

**Table 1:** Search strategy

Database	Search Terms	Search Field	Search Results
PubMed	(“cesarean section” OR “CS” OR “C-section” OR “cesarean delivery” OR “abdominal delivery”) AND (saline* OR NS OR “normal saline”OR “sodium chloride”) AND (irrigat* OR lavage OR “wound irrigat*” OR “wound infiltrat*” OR “subcutaneous irrigat*” OR “subcutaneous infiltrat*”)	All Fields	142
Cochrane	(“cesarean section” OR “CS” OR “C-section” OR “cesarean delivery” OR “abdominal delivery”) AND (saline OR NS OR “normal saline” OR “sodium chloride”) AND (irrigation OR lavage OR “wound irrigation” OR “wound infiltration” OR “subcutaneous irrigation” OR “subcutaneous infiltration”)	All Fields	119
Web of Science (WOS)	(“cesarean section” OR “CS” OR “C-section” OR “cesarean delivery” OR “abdominal delivery”) AND (saline* OR NS OR “normal saline”OR “sodium chloride”) AND (irrigat* OR lavage OR “wound irrigat*” OR “wound infiltrat*” OR “subcutaneous irrigat*” OR “subcutaneous infiltrat*”)	All Fields	279
SCOPUS	(“cesarean section” OR “CS” OR “C-section” OR “cesarean delivery” OR “abdominal delivery”) AND (saline* OR NS OR “normal saline”OR “sodium chloride”) AND (irrigat* OR lavage OR “wound irrigat*” OR “wound infiltrat*” OR “subcutaneous irrigat*” OR “subcutaneous infiltrat*”)	Title, Abstract, Keywords	181
Google Scholar	(“cesarean section” OR “C-section” OR “cesarean delivery” OR “abdominal delivery”) AND (saline* OR “normal saline”OR “sodium chloride”) AND (irrigation OR “wound irrigate*” OR “wound infiltrat*” OR “subcutaneous irrigate*” OR “subcutaneous infiltrat*”)	All Fields	The first 100 records

Eligibility Criteria

We included RCTs with the following PICO criteria: population (P), adult women undergoing either elective or emergency CS; intervention (I), SC saline irrigation; control (C), no irrigation; and outcomes (O), the incidence of wound complications (superficial SSI, hematoma, seroma, wound separation) and operative duration. Exclusion criteria comprised non-randomized trials, observational studies, in vitro laboratory studies, animal studies, posters, and conference abstracts.

Study Selection

Following duplicate removal using the Covidence online tool [17], four independent reviewers (LA, MA, AA, and HA) assessed each study twice for both screening and eligibility. The evaluation process was conducted in two stages. In the first stage, the reviewers separately screened the titles and abstracts of all identified articles to ascertain their relevance to this meta-analysis. In the second stage, they screened the full-text articles of the selected abstracts to determine their eligibility for inclusion. Any disagreements were resolved through discussion and consensus with others (WA, SA, and HH). This same process was applied during the methodological and statistical assessment.

Data Extraction

We drafted and pilot-tested an extraction sheet for the following data: study characteristics (country, study design, total participants, intervention, control, saline preparation method, CS type, primary outcome, main inclusion criteria, and follow-up duration); baseline characteristics (age, number of patients in each group, body mass index (BMI), gravidity, parity, fetal birth weight, diabetes mellitus, and smoking); and outcome data wound complications (superficial SSI, hematoma, seroma, wound separation) and operative duration. Any discrepancy was handled through discussion.

Risk of Bias

Two reviewers conducted an independent quality assessment to evaluate the risk of bias in the studies included, using the Cochrane Risk of Bias (RoB-II) tool [18]. This tool assesses multiple domains such as the randomization process, deviations from intended interventions, missing outcome data, measurement of outcomes, and selection of reported results. Each domain, along with the overall quality of the study, was categorized as low risk, some concerns, or high risk. Any discrepancies were resolved through discussion.

Statistical Analysis

RevMan version 5.4 (The Cochrane Collaboration, Oxford, UK) software was used to conduct the statistical analysis, implementing the random-effects model [19]. To combine the results of dichotomous outcomes, we utilized the risk ratio (RR), and for continuous outcomes, we employed the mean difference (MD), both with a 95% confidence interval (CI). Heterogeneity was assessed using the Chi-square and I-square tests. The Chi-square test evaluated the presence of heterogeneity, while the I-square test measured its extent. According to the Cochrane Handbook (chapter 9), we interpreted the I-square values as follows: not significant for 0-40%, moderate heterogeneity for 30-60%, and substantial heterogeneity for 50-90% [16]. We considered an alpha level below 0.1 for the Chi-square test to denote significant heterogeneity. In this study, we were unable to evaluate the presence of publication bias using Egger's test for funnel plot asymmetry. According to Egger and colleagues, assessing publication bias is unreliable when fewer than 10 studies are pooled [16].

Results

Search Outcomes and Selection of Studies

**Figure 1:**
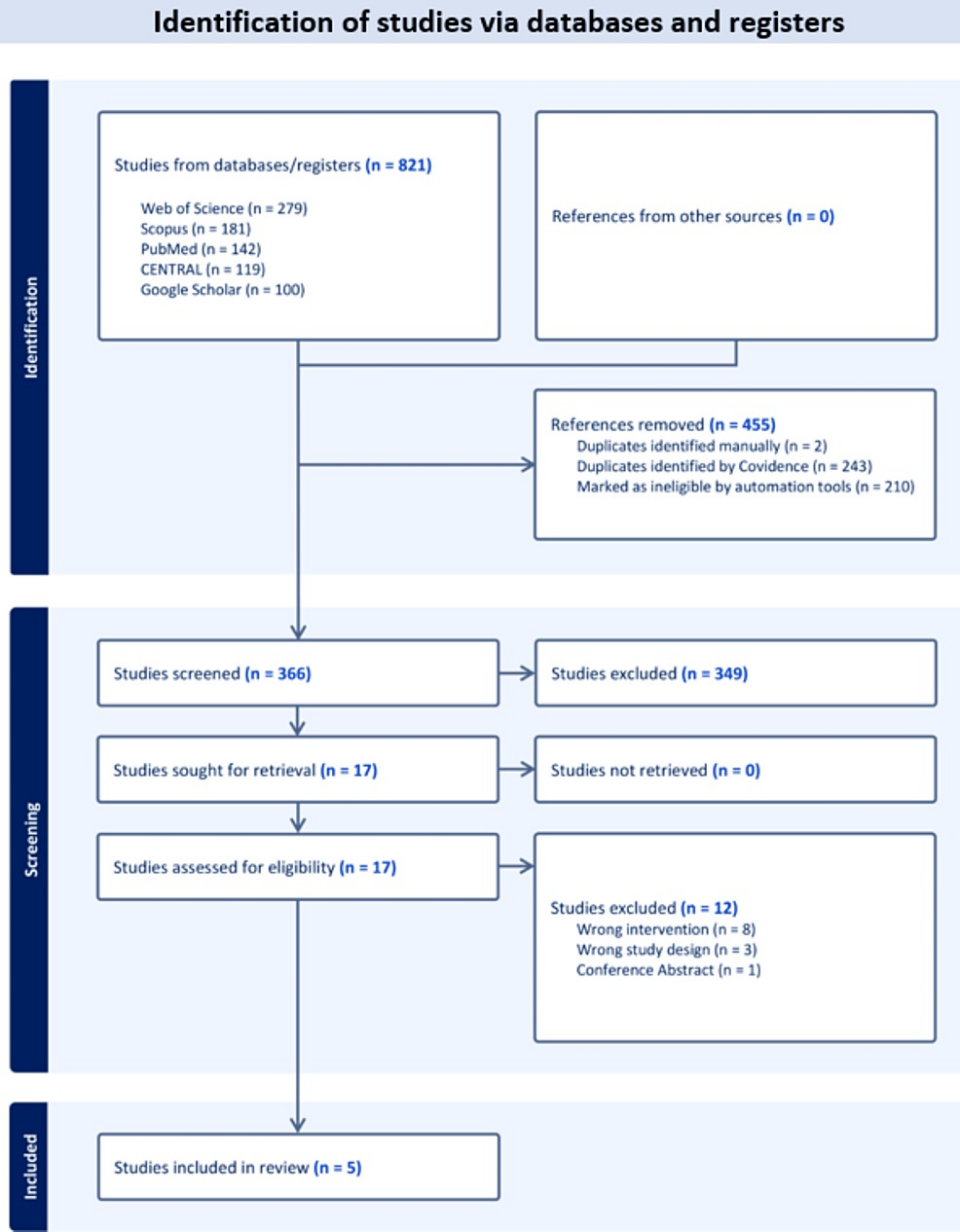
PRISMA flow diagram for the systematic search and study selection PRISMA: Preferred Reporting Items for Systematic Reviews and Meta-Analysis

We initially retrieved 821 records from our database search. Covidence automatically excluded 455 duplicates and ineligible studies. This left 366 records for title and abstract screening, of which 17 were selected for full-text screening. Ultimately, we included five studies [7,8,13,14,20] in qualitative and quantitative analysis (Figure 1).

Characteristics of Included Studies

We included five RCTs with 4.025 patients undergoing CS [7,8,13,14,20]. Four RCTs included patients undergoing elective CS only [7,8,13,14], while Güngördük et al. included patients undergoing elective or emergency CS [20]. The follow-up duration ranged from seven days to six weeks. Further details about the included studies and participants are outlined in (Tables 2-3), respectively.

**Table 2:** Summary of characteristics of the included trials NS: normal saline; CS: cesarean section; SC: subcutaneous; SSI: surgical site infection; N/A: not available

Study ID	Study Design	Country	Total Participants	Intervention	Control	NS Preparation Method	CS Type	Main Inclusion Criteria	Follow-Up Duration
Gomaa et al. (2022) [7]	Randomized controlled trial	Egypt	N=2890	SC saline irrigation	No irrigation	200 cc saline	Elective	20-40 years old women undergoing elective CS	30 days
Aslan Çetin et al. (2018) [8]	Randomized controlled trial	Turkey	N=185	SC saline irrigation	No irrigation	200 cc of saline (0.9% NaCl)	Elective	18-40 years old women undergoing CS for the first time, with no prior history of lower abdominal surgery	30 days
Mohamed et al. (2023) [13]	Randomized controlled trial	Egypt	N=200	SC saline irrigation	No irrigation	N/A	Elective	Obese women (body mass index >30 kg/m2, term pregnancy and singleton pregnancy	6 weeks
Gül DK (2021) [14]	Randomized controlled trial	Turkey	N=230	SC saline irrigation	No irrigation	200 cc of saline (0.9% NaCl)	Elective	20-40 years old women with a gestational age of 37-42 weeks, undergoing CS for the first or second time	7 days
Güngördük et al. (2010) [20]	Randomized controlled trial	Turkey	N=520	SC saline irrigation	No irrigation	100 cc of normal saline with a 30-60 mL syringe	Elective or emergency	Women past 37 weeks gestation	6 weeks

**Table 3:** Baseline characteristics of the participants SC: subcutaneous; N/A: not available; SD: standard deviation; N: number; DM: diabetes mellitus

Study ID	Number of Patients in Each Group		Age (Years), Mean (SD)		Gestational Age (Week), Mean (SD)		BMI, Mean (SD)		Fetal Birth Weight (g), Mean (SD)		Gravidity, Mean (SD)		Parity, Mean (SD)		DM, N (%)		Smoking, N (%)		
	SC saline	Control	SC saline	Control	SC saline	Control	SC saline	Control	SC saline	Control	SC saline	Control	SC saline	Control	SC saline	Control	SC saline	Control	
																			Gomaa et al. (2022) [7]	1445	1445	31.4 ± 6.3	31.3 ± 6.3	38.1 ± 1.4	38 ± 1.4	28.9 ± 4.3	28.5 ± 4.6	3477 ± 428	3362 ± 434	N/A	N/A	N/A	N/A	N/A	N/A	N/A	N/A	
Aslan Çetin et al. (2018) [8]	91	94	29.37 ± 6.13	28.76 ± 5.69	38.09 ± 0.80	38.06 ± 0.87	27.09 ± 2.76	26.80 ± 2.45	3206.92 ± 449.17	3296.60 ± 425.62	2.87 ± 1.22	2.97 ± 1.33	1.46 ± 1.04	1.59 ± 1.08	N/A	N/A	N/A	N/A	
Mohamed et al. (2023) [13]	100	100	26.44 ± 5.58	27.56 ± 4.79	N/A	N/A	33.84 ± 1.92	34.09 ± 1.92	N/A	N/A	3 ± 1.5	3 ± 1.5	1.67 ± 0.75	2 ± 1.5	N/A	N/A	N/A	N/A	
Gül DK (2021) [14]	115	115	30 ± 5.59	29 ± 5.15	38.1 ± 1.23	38.3 ± 1.75	30.72 ±8.77	30.66 ± 8.69	N/A	N/A	N/A	N/A	N/A	N/A	N/A	N/A	N/A	N/A	
Güngördük et al. (2010) [20]	260	260	26.25 ± 3.46	26.62 ± 3.82	39.12 ± 1.01	39.26 ± 2.23	30.88 ± 3.24	30.66 ± 3.13	N/A	N/A	N/A	N/A	N/A	N/A	2 (0.8)	3 (1.2)	17 (6.5)	13 (5)	

Risk of Bias

**Figure 2:**
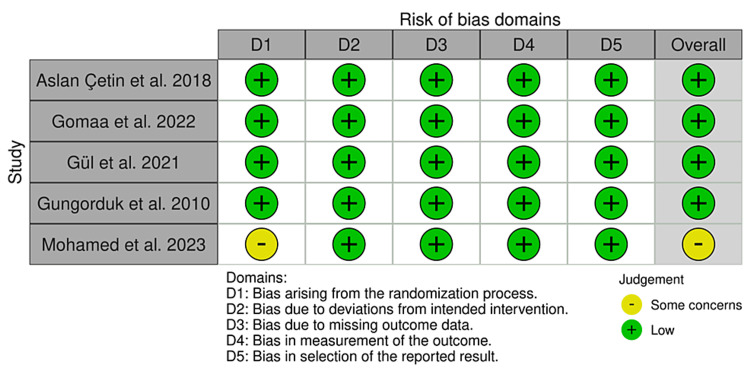
Risk of bias summary [7,8,13,14,20]

In general, the included RCTs exhibited a low risk of bias, except for Mohamed et al. [13] who lacked sufficient information on randomization. We assessed the "bias arising from the randomization process" domain as having "some concerns” (Figure 2).

Superficial SSI

**Figure 3:**
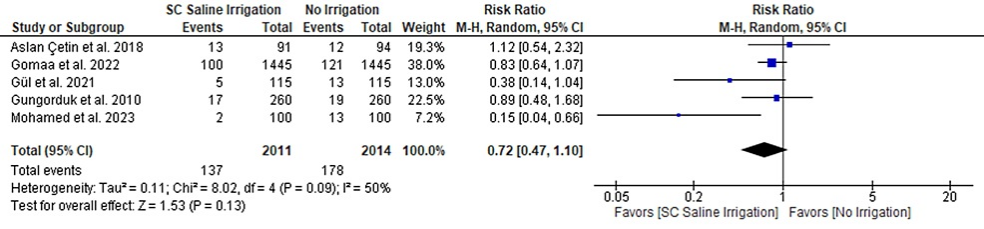
Meta-analysis of the rate of surgical site infection (SSI) [7,8,13,14,20] SC: subcutaneous

There was no substantial difference between SC saline irrigation and no irrigation regarding the incidence of superficial SSI (five RCTs, 4,025 patients; RR: 0.72 with 95% CI [0.47, 1.10], P = 0.13) (Figure 3). Pooled RCTs were inconsistent (P=0.09, I2= 50%).

Wound Complications

**Figure 4:**
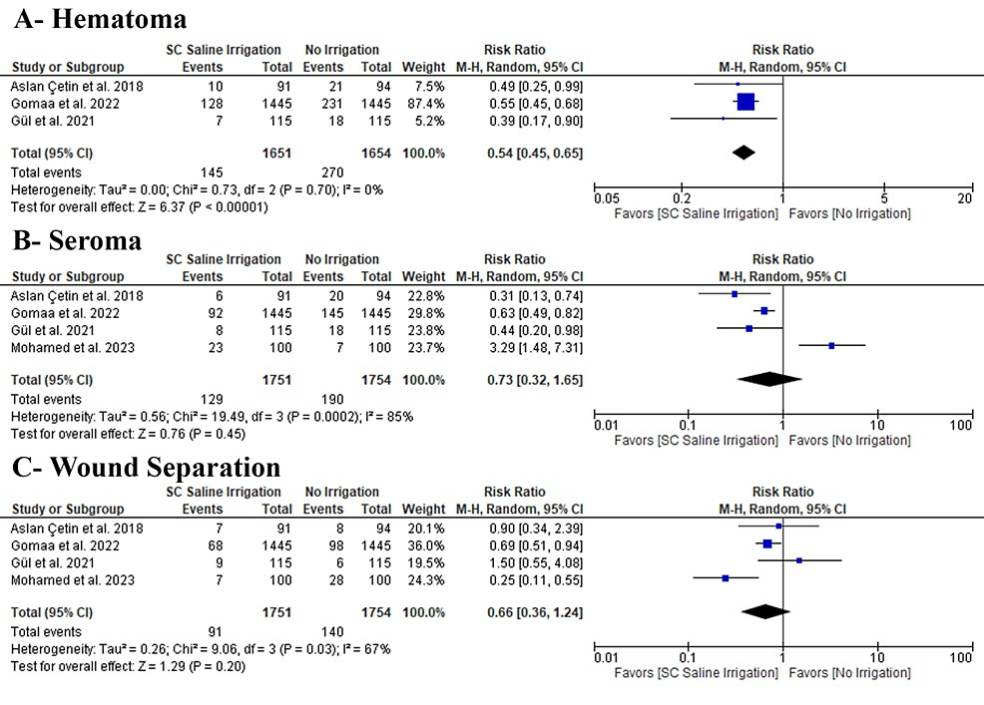
Meta-analysis of the rate of wound complications [7,8,13,14,20] SC: subcutaneous

SC saline irrigation significantly decreased the incidence of hematoma (three RCTs, 415 patients; RR: 0.54 with 95% CI [0.45, 0.65], P = 0.00001); however, there was no difference between SC saline irrigation and no irrigation regarding the incidence of seroma (four RCTs, 3,505 patients; RR: 0.73 with 95% CI [0.32, 1.65], P = 0.45) and wound separation (four RCTs, 3,505 patients; RR: 0.66 with 95% CI [0.36, 1.24], P = 0.2) (Figure 4). Pooled RCTs were consistent in hematoma (P = 0.7, I2= 0%); however, pooled RCTs were inconsistent in seroma (P = 0.0002, I2 = 85%) and wound separation (P = 0.03, I2 = 67%).

Operative Time

**Figure 5:**
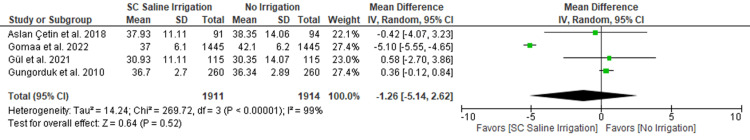
Meta-analysis of the mean change of operative time [7,8,14,20] SC: subcutaneous

There was no difference between SC saline irrigation and no irrigation regarding operative time (four RCTs, 3,825 patients; MD: -1.26 with 95% CI [-5.14, 2.62], P = 0.52) (Figure 5). Pooled RCTs were inconsistent (P = 0.00001, I2 = 99%).

Discussions

After synthesizing five RCTs, with 4,025 patients, SC saline irrigation after CS did not decrease the incidence of superficial SSI, seroma, or wound separation. However, SC saline irrigation decreased the incidence of surgical site hematoma. Also, there was no effect on the operative time.

The wound healing process is sequentially complete in two to four weeks from hemostasis, inflammation, epithelialization, fibroplasia, and maturation [7,8]. Any disruption of wound healing can lead to hematoma, seroma, wound separation, and eventually SSI. Hematoma can occur due to insufficient hemostasis, while seroma due to the inflammatory response, increasing exudation [7,21]. Subsequently, hematoma and seroma, which occur in 2-5% of women after CS, can lead to wound separation due to blood or serous collection, which opens a source to SSI [7,8]. This sequence is negatively impacting maternal recovery, prolonging hospital stay, and increasing healthcare costs [22].

Therefore, preventive measures have been investigated to prevent post-CS surgical site complications, including SC saline irrigation [7,8]. SC saline irrigation was reported to function by removing cellular debris, trapped fluids, or blood clots from the surgical site, which can stop the previously outlined pathological sequence [12]. However, our meta-analysis showed that SC saline irrigation only significantly prevented hematoma incidence. This can be due to the significant heterogeneity noted among the included studies. This can be supported by the significant effect of SC saline irrigation on seroma prevention after excluding Mohamed et al. [13].

After a bacteriological assessment of wound infections, Güngördük et al. reported that *Staphylococcus aureus* and *S. epidermidis* were the most common bacteria to contaminate the surgical site, regardless of SC saline irrigation [20]. Therefore, it can be speculated that SC wound irrigation can remove foreign materials or blood clots but it does not affect bacteria [20]. Thus, it had no effect on SSI prevention.

Furthermore, SC wound irrigation has been investigated in a heterogeneous manner so far, with different agents, including saline, antiseptics, and antibiotics [23]. Hence, the current evidence is not standardized with conflicting findings, which explains why SC wound irrigation has not been recommended in clinical guidelines so far [9]. Mueller et al. reported that SC irrigation significantly prevented SSI after abdominal operations [11]. However, he pooled various irrigation agents in his meta-analysis, and a subgroup analysis based on agents showed that only antibiotics prevented SSI, with no effect of saline irrigation [11], which is in line with our findings.

Strengths and Limitations

To the best of our knowledge, this is the first systematic review and meta-analysis to specifically examine the effect of SC saline irrigation after CS, and we only included data from RCTs, which represent the highest standard of evidence. However, our review has some limitations. Firstly, we only included trials from two developing countries (Egypt and Turkey), which may significantly affect the generalizability of our findings. Secondly, all the included trials involved patients undergoing elective CS under controlled conditions, with only Güngördük et al. [20] including both elective and emergency CS. Therefore, our data may not be applicable to emergency CS. Thirdly, all trials included patients with term pregnancies, limiting the data on patients undergoing pre-term CS, which are usually emergency procedures. Additionally, all outcomes except hematoma showed significant heterogeneity, which can be attributed to variations in patient characteristics or surgical measures. Finally, our review was not registered in the International Prospective Register of Systematic Reviews (PROSPERO) database.

Implications for Clinical Practice and Future Research

Given the lack of SC saline irrigation efficacy, institutional protocols are encouraged to implement evidence-based reliable, and feasible methods due to the increasing rates of CS and subsequently its complications [24]. These methods include routine preoperative antibiotics, antiseptic skin preparation, scissors usage instead of razors, povidone-iodine vaginal cleaning, suturing the SC tissue if its thickness is more than two centimeters, skin closure with sutures instead of closing with staples, removing dressing during 24 to 48 hours postoperatively, and using chlorhexidine gluconate soap after dressing removal [10,25,26].

Moreover, evidence of the head-to-head efficacy of SC irrigation with saline, antibiotics, or antiseptics is lacking. Thus, future RCTs are required to determine the most effective SC irrigation agent. Also, future RCTs should consider including patients undergoing emergency CS.

## Conclusions

After synthesizing five RCTs, with 4,025 patients, SC saline irrigation after CS did not decrease the incidence of superficial SSI, seroma, or wound separation. However, SC saline irrigation decreased the incidence of surgical site hematoma. Also, there was no effect on the operative time. Institutional protocols implementing multi-layer procedures are required to prevent SSI and further trials conducting head-to-head efficacy of SC irrigation with saline, antibiotics, or antiseptics are warranted.
